# Hybrid models combining trend and seasonality components with machine learning algorithms provide accurate forecasting of malaria incidence

**DOI:** 10.1371/journal.pgph.0004500

**Published:** 2025-10-17

**Authors:** Syed Shah Areeb Hussain, Sanchit Bedi, Chander Prakash Yadav, Ajeet Kumar Mohanty, Kalpana Mahatme, Suchi Tyagi, N. M. Anoop Krishnan, Sri Harsha Kota, Amit Sharma

**Affiliations:** 1 Academy of Scientific and Innovative Research (AcSIR), Ghaziabad, Uttar Pradesh, India; 2 ICMR—National Institute of Malaria Research (NIMR), New Delhi, India; 3 Department of Civil Engineering, Indian Institute of Technology (IIT) Delhi, Delhi, India; 4 ICMR—National Institute of Cancer Prevention and Research (NICPR), Noida, Uttar Pradesh, India; 5 State Programme Officer, National Center for Vector Borne Disease Control, NCVBDC, Directorate of Health Services (DHS), Panaji, Goa, India; 6 International Centre for Genetic Engineering and Biotechnology (ICGEB), New Delhi, India; Nanyang Technological University, SINGAPORE

## Abstract

Forecasting malaria incidence is vital for effective resource allocation during malaria elimination. In this study, we highlight robust models for forecasting incidence using climatic and malaria data from Goa, India. Multi-collinearity and Shapley Additive Explanations (SHAP) were used to identify most important predictors of malaria transmission among 15 climatic variables. Three machine-learning models (Support vector machines, Random Forest, Extreme gradient boosting), three time-series models (ARIMA, SARIMA, SARIMAX), and three hybrid models (RF-ARMA, SVM-ARMA, XGB-ARMA) were then trained and tested on data spanning from 2010 to 2019. Climatic extremes have stronger influence on malaria transmission than average values in Goa. Machine learning models exhibit lower accuracy (Root Mean Square Error (RMSE):13–37) but high precision (lower confidence intervals). Conversely, time series models, yielded more accurate results (RMSE: 5–41) albeit with less precision (wider confidence interval). To address this, we augmented machine learning models by incorporating time series variables which significantly bolstered their accuracy while retaining their inherent precision (RMSE: 0**·**5-15). Integrating time-series components into machine learning models harnesses the strengths of both approaches resulting in a substantial enhancement in accuracy and precision of forecasts. This technique has potential for wider use in planning malaria elimination, and routine epidemiological data analysis.

## Introduction

Having failed to eradicate malaria in the 1960s, the world is once again vying for malaria elimination. Several countries have successfully eliminated indigenous transmission of malaria in the last two decades. However, traditional malaria control strategies are resource intensive due to blanket application of interventions and fail to account for emerging challenges such as insecticide resistance. Hence, there is a need for a more proactive approach with targeted interventions.

Data-driven forecasting plays a pivotal role in infectious disease epidemiology by helping public health officials and policy makers make informed decisions for targeted control and mitigation. Biological models for infectious diseases such as cholera, small pox, and measles have been around since the 18^th^ century, though the foundations of mathematical epidemiology were first laid down by Ronald Ross through his dynamic malaria model [1]. Biological models are dependent on the assumptions of the underlying mechanisms involved in biological systems, due to which they are inherently difficult to model and forecast [2]. Hence, they perform better for emerging infections with less data than for established infections such as malaria [2]. Consequently, in recent years, attempts at forecasting malaria have shifted towards empirical approaches which include regression based techniques that analyze relationships with environmental predictors [3,4] or time-series models that use patterns and trends in historical data to make predictions [5,6]. More recently, models specifically designed for machine learning, that can automatically recognize patterns in data and improve with experience, have been used for forecasting malaria [7,8]. However, the predictive accuracy of different types of models varies significantly in different studies and across different regions.

Climatic variables are often used as proxies to simulate vector development and abundance when modelling malaria transmission. The effect of temperature on malaria is well documented, with transmission limited by higher temperatures in some regions [9] and lower temperatures in others [10]. Rainfall also directly affects malaria transmission through creation of breeding sites [11]. Strong positive association between relative humidity and mosquito behaviour has also been observed in some studies [12–14]. Impact of atmospheric pressure, and wind speed on malaria transmission is less perceptible, though several studies have shown significant associations [8,15]. Factors such as land use/land cover and vegetation can also affect transmission, but do not vary on shorter timescales and therefore are more relevant for comparing transmission across different locations than for short-term temporal forecasting. The role of different environmental predictors in malaria transmission also varies significantly across regions [6].

Forecasting malaria incidence has compelling public health significance for a large country like India where effective resource allocation is critical [16]. The present study aims to assess the comparative performance of different models in their ability to make accurate short-term forecasts of malaria. The study uses data from the coastal state of Goa, which has recently entered the elimination phase for malaria control. The outcomes of this study will provide valuable insights for designing targeted interventions, optimizing resource allocation, and strengthening early warning systems, thereby aiding the development of evidence-based strategies and policies for improved malaria control in India.

## Methods

### Study site

**Fig 1:**
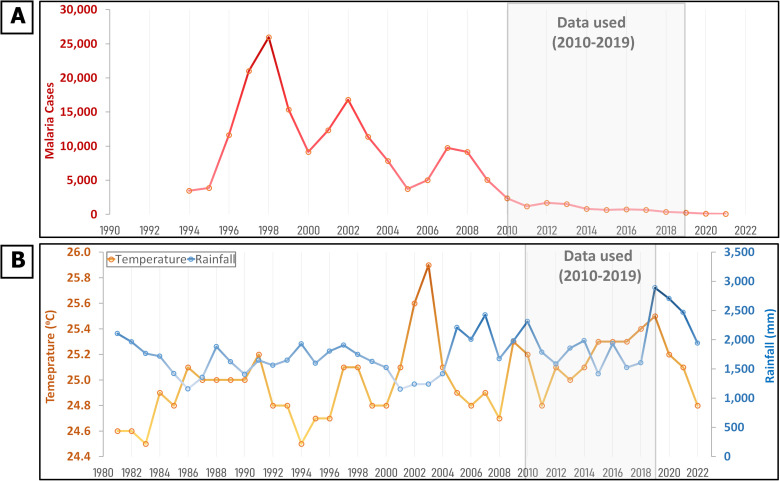
Long term trends in malaria and climatic features in Goa. (A) Trend of malaria cases in Goa since 1994 and (B) temperature and rainfall trend in Goa since 1980.

Goa, situated on India’s western coast, experiences a tropical monsoon climate (Amw) [17] Monthly temperatures range from 25 - 32 °C, with maximums not exceeding 35 °C. Winter temperatures usually don’t fall below 20 °C. The monsoon season spans June to September with annual rainfall of 2800–3800 mm (Fig 1B). Goa is malaria-endemic due to its favourable climate, and prior to 2010 over 8000 cases were reported annually (Fig 1A). Since then the burden has reduced substantially (<1000 cases annually), with no malaria-related deaths since 2018 [18]. Therefore, it was classified under the malaria elimination phase in the National Strategic Plan for Malaria Elimination 2017 [16]. Making accurate forecasts under this declining trend can be particularly challenging when quantifiable data on interventions is not available, therefore, Goa serves as a relevant case study for our study. The state comprises two districts: North Goa and South Goa, with the former bearing a higher malaria burden.

### Data sources

Monthly malaria incidence data for North and South Goa, from 2010 to 2019 were obtained from the National Center for Vector borne Disease Control (NCVBDC). Data for more recent years (2020–2021), was excluded to eliminate bias resulting from lapses in surveillance during the COVID-19 pandemic lock-downs. Monthly data of maximum (max.), minimum (min.), and average (avg.) (total for rainfall) of five meteorological variables - temperature, rainfall, relative humidity, atmospheric pressure, and wind speed, was extracted from the ERA5 dataset [19], a reanalysis dataset that applies physical principles and combines model data with worldwide observations using data assimilation, wherein an earlier forecast is optimally merged with recently acquired observations at regular intervals.

### Data pre-processing and exploratory analysis

Short-term monotonous trends in the datasets were analyzed using the seasonal Mann-Kendall test. Seasonal plots were used to analyze variable seasonality while scatter plots revealed the relationship between meteorological variables and malaria cases. Correlation plot of a predictor variables was used to identify multi-collinearity (Pearson’s Correlation (r) > 0**·**7). To address bias due to linearity assumptions, one-parameter standard Box-Cox transformation was applied on all variables for the time series models, which applies power transformation based on the equation 1:


$$y(\lambda )=\frac{y^{\lambda }-\text{1}}{\lambda },\;if\;\lambda \neq \text{0};$$

        = logy,   if λ=0; (1)

where λ is the transformation parameter determined based on the Guerrero method, which selects λ that minimizes coefficient of variation of the transformed data. This transformation also prevents negative predictions of malaria cases in the time-series models, which are likely given the significant downward trend of incidence. Data was standardized for the machine learning and hybrid models by subtracting by the mean and dividing by the standard deviation.

### Feature selection

Initially three preliminary models – random forest (RF), support vector machines (SVM), and extreme gradient boosting XGB) were trained using all fifteen predictors, and optimized using grid search with up to 50 configurations. Of the three models, XGB had the lowest error rates in both the training and testing dataset and was therefore used for feature selection. Shapley additive explanations (SHAP) were used to interpret the outputs and identify the feature contributions in the best-performing model (i.e., XGB). For each pair of collinear variables (high correlation) the one with the lower SHAP score was eliminated, resulting in six final predictors – max. temperature, min. temperature, total rainfall, avg. relative humidity, min. atmospheric pressure, and min. wind speed.

### Data partitioning for training and testing

The original time-series dataset was split into two training and testing datasets, used to build the model and assess performance respectively. The first dataset used data from 2010-2017 for training and data from 2018 for testing, while the second used data from 2010-2018 for training and data from 2019 for testing. This allowed comparison of forecasts for two different years, capturing the declining trend of malaria in the state [20].

### Model building and optimization

Planning and implementation of intervention efforts for malaria control in India currently occurs at the district level. Models were, therefore, trained separately for North Goa and South Goa so that the results were meaningful to the current malaria control and elimination efforts. Initially, three Machine Learning models and three time series models were built and analyzed, after which additional hybrid models were built which incorporated aspects of both machine learning and time series models (Fig G in S1 Text). All models were built using the R version 4.3.1 [21] within the RStudio graphical user interface [22].

#### Machine learning models.

Three machine learning models RF, SVM, and XGB were used for forecasting malaria in Goa. RF uses a bagging algorithm to fit various subsets of data over multiple decision trees. Repeated cross-validation with 10 folds and three repeats was used for tuning the mtry and ntree hyperparameters based on a grid search algorithm (50 hyperparameter configurations). Mtry controls number of features at each split and ntree defines the total number of decision trees.

SVM regression is a supervised machine learning algorithm that finds the best hyperplane that fits the input data in a continuous space. Grid search was used to tune gamma and cost parameters with 100 different configurations. These control localization of training sample and trade-off between overfitting and underfitting respectively. Bootstrapping (500 iterations) was used to generate confidence intervals for the model.

XGB is another tree-based ensemble learning algorithm that uses boosting instead of bagging, i.e., trees are built sequentially, optimizing loss function after each tree. Hyperparameters max. depth of trees, number of trees, learning rate, min. loss reduction, proportion of training sampled, and the minimum sum of instance weights in a child node were tuned by grid search using repeated cross validation with 10 folds and three repeats. Bootstrapping (500 iterations) was used to generate confidence intervals for the model.

#### Time-series models.

Three time-series forecasting models were used in the study – ARIMA, SARIMA and SARIMAX model. ARIMA model combines AR - autoregressive (linear combinations of past values), I - differencing (stabilizes mean of a time series), and MA - moving average (weighted moving average of past forecasting errors) components to capture patterns and trends in time-series data. SARIMA is an ARIMA model that includes seasonal period specific AR and MA terms. SARIMAX stands for seasonal ARIMA with exogenous variables and includes external predictors that are outside the control of the time-series but may still influence it. The auto.arima function in the R forecast package was used to determine the optimal order of the AR, MA, and I terms by comparing AIC values from 200 iterations of each model. Models with similarly low AIC values were selected and their confidence intervals and error rates in forecasts were compared to select the best ARIMA, SARIMA, and SARIMAX model out of all the different iterations.

#### Machine learning models with auto-regressive and moving-average terms.

The final set of models combined both the machine learning and time-series models. This was done by first fitting a SARIMA model to the malaria case time-series testing up to 20 lags. The seasonal and non-seasonal AR order (P, p) were then extracted from this model to generate lagged response variables for each lag. Additionally, the one-step residuals were also extracted from the SARIMA model and used as proxies for the latent error dynamics associated with the seasonal and non-seasonal MA components. These SARIMA derived features were then combined with the climatic predictors and used to train the three machine learning models (RF, SVM, and XGB). A walk-forward rolling algorithm was applied such that after each forecast, the SARIMA model was updated with the new generated response predictions and the time-series features were recomputed for the next forecast. Hyperparameter tuning and model optimization steps were kept the same as the initial machine learning models.


$$\hat{y_{t}}=\;f\left(\underset{\;if\;p>\text{0},\;else\;empty}{\underbrace{y_{t-\text{1}},\;.\;.\;.\;,\;y_{t-p}}},\underset{if\;P>\text{0},\;else\;empty}{\underbrace{y_{t-s},\;.\;.\;.\;,\;y_{t-sP}}},\underset{if\;q>\text{0},\;else\;empty}{\underbrace{\varepsilon_{t-\text{1}},\;.\;.\;.\;,\;\varepsilon_{t-q}}},\underset{if\;Q>\text{0},\;else\;empty}{\underbrace{\varepsilon_{t-s},\;.\;.\;.\;,\;\varepsilon_{t-sQ}}},\;X_{t}\right)$$

where *y* represents the response variable, *t* represents the current time-step in the algorithm, s is the seasonal frequency (12 months), *p* is the non-seasonal AR order, *q* is the non-seasonal MA order, *P* is the seasonal AR order, *Q* is the non-seasonal AR order, ε is the residual and *X*_*t*_ is the set of climatic covariates. To compute the confidence intervals for these models, 50 iterations of the time-series models were run and the auto-regressive and moving average features were recomputed at each iteration and passed over to the machine learning models. The machine learning models were then retrained for each of these iterations using the original bootstrapping algorithm. This was done so that the uncertainty in the time-series model outputs could be reflected in the final models based on the new time-series features computed at each step.

### Validation of predictions

Model performance was compared using error based metrics, namely – the mean absolute error (MAE) and the root mean square error (RMSE). The forecasts were plotted against the actual case incidence obtained from surveillance and the width of the confidence intervals for each of the forecasted value was also compared to assess the reliability of predictions. Wider confidence intervals indicate a higher degree of uncertainty in the predictions, whereas narrow confidence intervals indicate higher precision in predictions. Significance of differences in the forecasts of different models was assessed based on pair-wise Deibold-Mariano tests.

## Results

### Seasonality and trends in the environmental variables and malaria in Goa

Seasonal plots of environmental variables (Fig A & B in S1 Text) show that avg. monthly temperature in Goa range between 25–30 °C, while max. and min. monthly temperatures are 35 °C and 15 °C respectively. Rainfall is primarily concentrated between June and September. Max. relative humidity remains consistently high, exceeding 80% through most of the year, except in drier months from November to March. Pressure variables exhibit a gradual drop from late winter to monsoon (June-July), followed by an increase until mid-winter (December). Only min. wind speed showed significant seasonality with higher min. wind speeds in the monsoon season. The malaria season typically spans from May to November, with two peaks observed in several years (July/August and October/November), corresponding to low max. temperatures, moderate rainfall, high avg. relative humidity, and high min. wind speeds (Fig A & B in S1 Text).

Seasonal Mann-Kendall test reveals declining trend in malaria cases since 2010, likely due to increased intervention efforts (Table A in S1 Text). All three temperature parameters show strong positive trend across both North Goa and South Goa, a possible indication of climate change. Additionally, significant positive trends are also observed in min. RH, max. pressure, and avg. pressure. In contrast, min. rainfall exhibits a significant declining trend, suggesting an increase in drought-like conditions. The min. wind speed also shows a significant declining trend in the two districts.

### Selection of the best predictors

Of 15 meteorological variables, min. temperature, max. rainfall, total rainfall, min. RH, avg. RH, and min. wind speed show significant positive association with malaria cases, whereas the variables avg. temperature, max. temperature, min. pressure, and avg. pressure demonstrate significant negative association (Fig C & D in S1 Text).

SHAP analysis reveals that total rainfall has the strongest impact on model outputs, with an effect size more than twice that of any other parameter. Furthermore, avg. variables were found to have low impact on model outputs, whereas the min. and max. variables significantly affected model performance (Fig F in S1 Text). Among the top 10 parameters with highest contribution to model outputs (Median SHAP value > 1), significant correlations were observed between - min., max., and avg. pressure variables (0·8-0·95), total and max. rainfall variables (0·72), and avg. and min. relative humidity variables (0·85) (Fig E in S1 Text). Of these variables with higher median SHAP value were retained, resulting in the final set of predictors: max. temperature, min. temperature, total rainfall, avg. RH, max. pressure, and min. wind speed.

### Forecasts of malaria incidence using machine learning models

Among the three machine learning models, XGB outperformed both RF and SVM for both North Goa and South Goa (Table 1), with lower MAE and RMSE. The forecasts for 2018 and 2019 were relatively comparable in North Goa, whereas in South Goa, forecasts for 2018 were much more accurate than for 2019.

**Table 1:** Error rates (RMSE & MAE) of malaria forecasts in North Goa and South Goa for the years 2018, 2019, 2020 and 2021 using three different machine learning models (SVM, RF and GB).

		North Goa		South Goa	
		2018	2019	2018	2019
**SVM**	RMSE	27·73	30·38	18·99	30·44
	MAE	23·09	25·92	13·19	29·75
**RF**	RMSE	36·67	32·59	18·23	26·67
	MAE	30·98	28·30	14·87	22·85
**XGB**	RMSE	21·28	25·05	18·12	25·46
	MAE	17·68	21·19	13·16	23·42
**ARIMA**	RMSE	28·23	14·51	14·68	8·43
	MAE	19·85	11·96	12·75	7·74
**SARIMA**	RMSE	14·99	16·99	9·88	8·38
	MAE	11·08	12·82	7·08	5·24
**SARIMAX**	RMSE	40·94	16·85	16·57	8·24
	MAE	32·61	8·31	14·03	6·08
**SVM-ARMA**	RMSE	0·72	1·07	9·01	6·71
	MAE	0·54	0·74	6·58	5·09
**RF-ARMA**	RMSE	7·35	11·37	9·01	14·86
	MAE	5·67	8·01	7·10	10·74
**XGB-ARMA**	RMSE	5·87	3·55	8·74	3·02
	MAE	4·41	2·71	6·69	2·27

**Fig 2:**
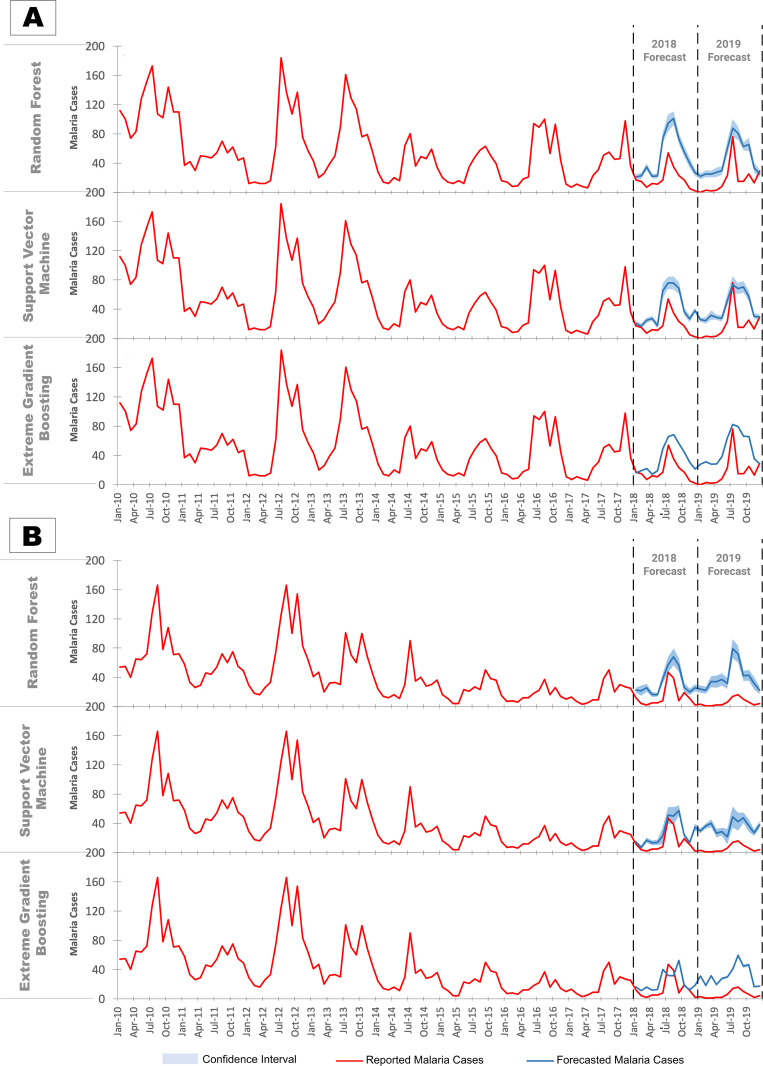
Forecasts of malaria against reported malaria cases based on machine learning models in (A) North Goa and (B) South Goa.

In North Goa all three machine learning models significantly overpredict malaria cases in 2018 (Fig 2A). However, the higher peak of malaria cases in 2019 is captured more accurately. Nonetheless, the forecasts still exceed the actual case counts significantly for other months.

Conversely, in South Goa, forecasts were more accurate in 2018 than in 2019 (Fig 2B). The models failed to capture the decline in malaria cases in 2019, and tend to over predict malaria incidence, resulting in less accurate forecasts compared to 2018. Overall, forecasts of the XGB and SVM model did not differ significantly from each other, but were significantly different from those of the RF model (Table N in S1 Text).

### Forecasts of malaria incidence using time-series models

The time series models showed significant heterogeneity in error rates for the forecast years 2018 and 2019 (Table 1). SARIMA performed best for forecasts in 2018 for both North and South Goa. SARIMAX model forecasts had lower error rates in 2019 for North Goa. However, in South Goa all three models showed similar error rates in 2019 forecasts. The forecasts of the SARIMAX model in South Goa were not significantly different from the ARIMA model (Table N in S1 Text), and the SARIMA model appeared to be the best-performing model here. Overall time series models had lower error rates than machine learning models, indicating greater forecasting accuracy.

**Fig 3:**
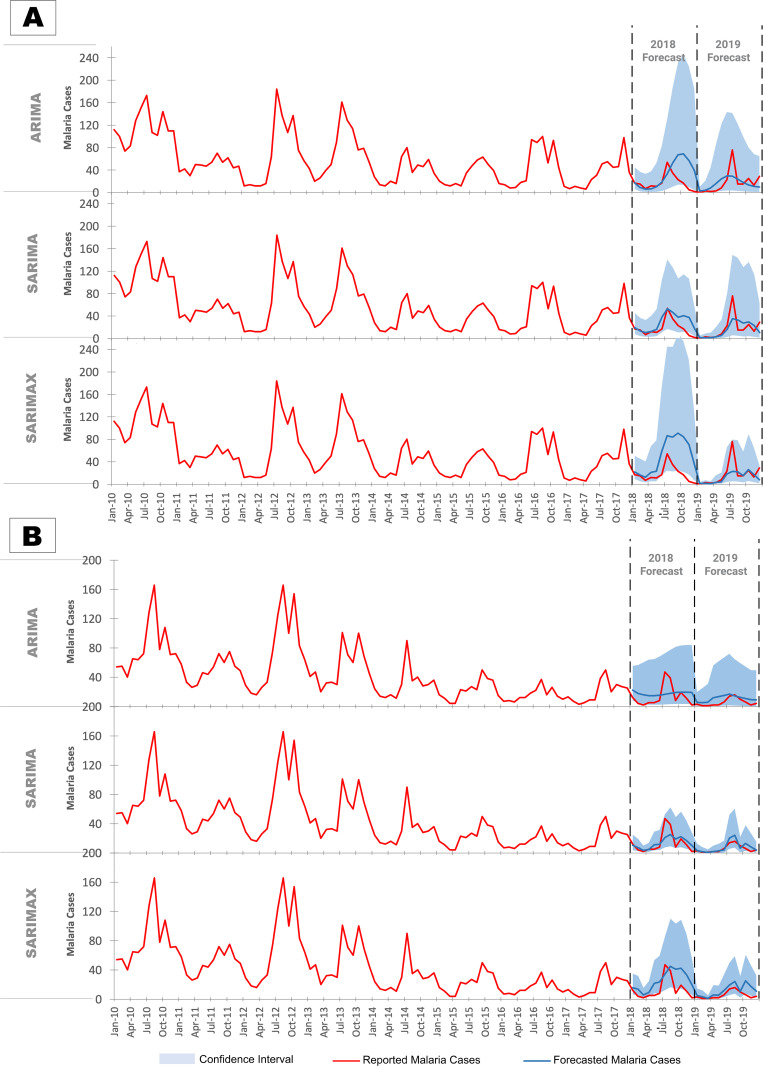
Forecasts of malaria against reported malaria cases based on time series models in (A) North Goa and (B) South Goa.

Fig 3A illustrates the high accuracy of SARIMA in forecasts for 2018 in North Goa, closely following the actual malaria case counts, while ARIMA and SARIMAX slightly overpredicted. Contrastingly, all three models significantly underestimate cases in North Goa for 2019, relying heavily on the declining trend in malaria incidence over the years, not accounting for the anomalous spike in incidence.

This is more clearly demonstrated in South Goa, where no spike in malaria cases occurred in 2019, and models accurately captured the declining trend in cases (Fig 3B). Time series models, therefore, struggle with anomalies in data, even when external predictors are incorporated. Furthermore, these predictions have low reliability reflected by the wide confidence intervals.

### Forecasting using machine learning models with time series features

To address the trade-off between accuracy and precision between machine learning and time series models hybrid ML-ARMA models were built. The order for the seasonal and non seasonal AR and MA terms identified by the SARIMA model were p = 1, P = 0, q = 1 and Q = 1 respectively. Addition of time series features to machine learning models greatly improved the forecast accuracy for both 2018 and 2019, with extremely low error rates in both the training (Table D in S1 Text) and testing datasets (Table 1). Among the three hybrid models, forecasts were most accurate for the SVM-ARMA model in North Goa and XGB-ARMA model in South Goa.

**Fig 4:**
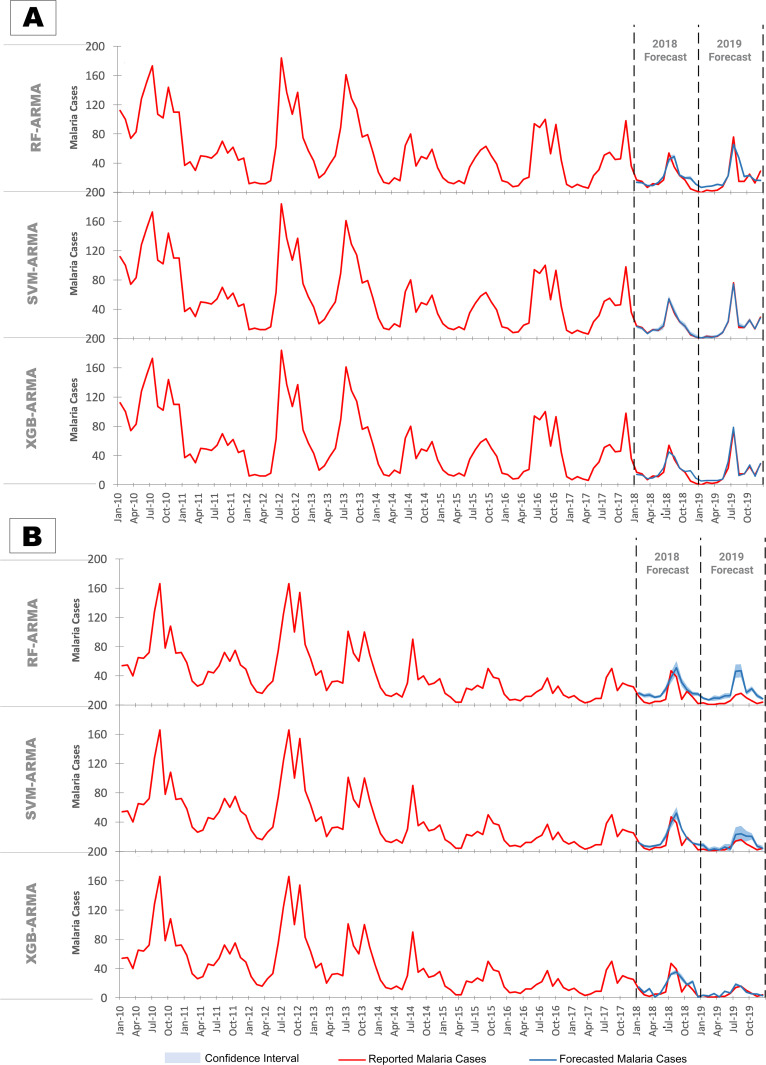
Forecasts of malaria against reported malaria cases based on hybrid ML-ARMA models in (A) North Goa and (B) South Goa.

Plots of model forecasts reveal strong agreement of the SVM-ARMA and XGB-ARMA models with observed cases in North Goa (Fig 4A). All models accurately predicted the higher peak of malaria cases in 2019, indicating their ability to handle anomalies.

In South Goa, forecasts were slightly less accurate, yet the models successfully captured the peak in 2018. SVM and RF forecasts were slightly higher, while XGB forecasts were marginally lower than reported cases (Fig 4B). Forecasts for 2019 revealed that RF model missed the downward, SVM overpredicted, and XGB almost perfectly matched the actual reported incidence. Overall, the XGB model with time series features emerged as the best choice, offering fairly accurate predictions for North Goa and superior predictions for South Goa. The forecasts of this model show significant difference from all the other models tested (Table N in S1 Text), in addition to the lowest error rates.

## Discussion

To achieve malaria elimination by 2030 in India, a country with diverse climate and ecology, leveraging all available technologies is crucial. Malaria forecasting is an effective tool for efficient resource allocation and support early detection and preparedness. In this study we compare models for short term forecasting (one year in advance) of malaria cases in Goa, a representative where the overall malaria incidence has declined rapidly in the past decade.

Descriptive analysis of meteorological variables in Goa reveal that max. monthly temperature rarely rising above 32°C or dropping below 20°C. These ambient conditions favor vector growth year-round, with monsoon months (avg. temperature 26°C - 29°C) being most suitable for the major malaria vector in Goa, i.e., *Anopheles stephensi*. Consequently, max. and avg. temperature correlates negatively with malaria cases, while min. temperature correlates positively. Similarly relative humidity rarely falls outside the tolerance range of *An. stephensi* (50–80%). Most of the rainfall is concentrated between May-October and can be a major limiting factor for malaria transmission. Excess rainfall during July and August may even result in flushing of vector habitats leading to reduced malaria transmission in subsequent months. Consequently, Goa often experiences two peaks in malaria cases. Total monthly rainfall was found to be the single largest contributor to malaria cases in our analysis, with an effect size more than twice as much as any other variable. Wind and pressure also strongly influenced model performance. Wind may affect dynamics of malaria transmission by influencing Anopheline populations through wave activity, advection of adult Anopheline mosquitoes, and CO2 attraction related host-seeking behaviour of mosquitoes [15]. Overall, average factors have a lesser impact on the model performance as compared to the min. and max. values.

Our study found that machine learning models overestimated the malaria incidence in Goa, but were better at predicting outbreaks (such as in North Goa in 2019) due to their reliance on external predictors, which enables them to predict outside previously observed trends. Furthermore, these predictions have narrow confidence intervals indicating high precision (high bias, low variance). In contrast, time series models had slightly lower RMSE and MAE values indicating higher accuracy, but were not able to predict anomalies, i.e., in North Goa in 2019. Moreover, they were plagued with fairly wide confidence intervals resulting in low precision (low bias-high variance). Numerous studies have assessed the comparative performance of these models in forecasting malaria incidence in different parts of the world. While machine learning models usually out-perform the time-series models in forecasting disease incidence [23–26], the reverse has also been observed in some scenarios, particularly when the data is limited and shows strong linearity [20,27–29]. To address this trade-off, recent attempts at infectious disease forecasting have shifted towards combining multiple models, so that the strengths and weaknesses of each individual model can harnessed. Ensemble methods involve aggregating the outputs of multiple models using simple averages, weighted average or ranking, and these have shown to significantly improve upon the forecasts of individual models [30–32]. Stacking methods use a meta-learner model to combine multiple model outputs and were found to be even superior to other ensemble techniques [33,34]. Zhang et al. [35] on the other hand, adopted a sequential combination technique which administered models step by step based on data characteristics [35]. These specialized hybrid models have gained prominence recently, owing to their ability to model the linear and non-linear components of the data individually, and have shown high accuracy in forecasting disease incidences [29,35–38]. However, these hybrid models have so far not been applied to malaria incidence in India. Therefore, to leverage the benefits of both time-series and machine learning models, we built hybrid ML-ARMA models that combine time series features with machine learning algorithms. Malaria datasets generally also include multiple cases related recrudescence and relapse, in addition to new infections. Coupled with the role of immunity during periods of high endemicity, information of previous infections are likely to significantly improve the performance of the models. Therefore, a SARIMA model was used to create lagged autoregressive and moving average features, which were incorporated within the machine learning algorithms to create the hybrid ML-ARMA models. A similar approach has been used by Martineau et. al. to combine lagged sea surface temperature features from a distributed log-linear model to machine learning algorithms to create highly accurate forecasts [30]. The hybrid ML-ARMA model show very high accuracy and precision, with 5–10 times lower error rates than the individual models. The XGB-ARMA model was found to be the most suitable, with reasonably accurate and precise forecasts in both North Goa and South Goa.

Limitations of our study include exclusion of mathematical models, absence of lagged predictors, and lack of variables like vegetation, urbanization, immunity, migration and interventions. Mathematical models are highly complex, depending on a range of different variables that are not easy to collect or measure and were hence excluded. Initial comparison of seasonal plots did not reveal any lagged effect of predictors, possible due to the perennial suitability for malaria in Goa. Urban clustering of malaria cases is indeed evident in Goa, with six times more malaria infections in migrant urban workers as compared to native Goans [39,40]. However, information regarding variations due to factors such as immunity and interventions are visible in historical trends of the malaria cases and vegetation and urbanization factors vary over more larger time-scales to have any significant effect on our model. Lastly, these models have been tested only in the coastal state of Goa, and a more comprehensive analysis in several different regions with varying levels of endemicity and climatic/environmental conditions may provide a more conclusive assessment.

## Conclusion

The results of our study provide a framework for developing accurate short-term forecasting models for malaria. Firstly, we show that climatic variables can provide sufficiently accurate predictions for malaria, given that a suitable feature selection method is applied to identify the limiting factors. Secondly, we show that history-informed machine learning models can account for trends and seasonality in data, and when combined with environmental predictors provide significantly more accurate and precise forecasts for malaria. The forecasting models thus produced in this study have sufficient accuracy and precision so that they may be used for effective resource allocation and outbreak warning in the national malaria control programme.

## Supporting information

S1 Text Supplementary material. (DOCX)
