# Supplementary material for: Hybrid models combining trend and seasonality components with machine learning algorithms provide accurate forecasting of malaria incidence
Source: PLOS Glob Public Health. 2025 Oct 17;5(10):e0004500. doi: 10.1371/journal.pgph.0004500 (PMC12533842; doi:10.1371/journal.pgph.0004500)
Supplement: S1 Text — (DOCX) [file pgph.0004500.s001.docx]

**History Informed Machine Learning models outperform conventional methods in Forecasting malaria incidence in Goa, India**

**Supplementary Material**

Syed Shah Areeb Hussain^1,2^, Sanchit Bedi^3^, Chander Prakash Yadav^4^, Ajeet Kumar Mohanty^2^, Kalpana Mahatme^5^, Suchi Tyagi^1,2^, N. M. Anoop Krishnan^3^, Sri Harsha Kota^3^ and Amit Sharma^1,6*^,

1. Academy of Scientist and Innovative Research (AcSIR), Ghaziabad, UP, India
2. ICMR – National Institute of Malaria Research (NIMR), New Delhi, India
3. Department of Civil Engineering, Indian Institute of Technology (IIT) Delhi, India
4. ICMR - National Institute of Cancer Prevention and Research (NICPR), Noida, UP, India
5. State Programme Officer, National Center for Vector Borne Disease Control, NCVBDC, Directorate of Health Services (DHS), Panaji, Goa, India
6. International Centre for Genetic Engineering and Biotechnology (ICGEB), New Delhi, India

***** Corresponding Author

Dr. Amit Sharma

amit.icgeb@gmail.com

**Table of Contents**

[Seasonality Plot of Malaria cases and Climatic Predictors 4](#_Toc208569579)

[**Figure A:** Seasonal plots of all fifteen predictor variables and malaria cases in North Goa. The grey colour indicates the known optimum range of these predictors for malaria transmission. 4](#_Toc208569580)

[**Figure B:** Seasonal plots of all fifteen predictor variables and malaria cases in South Goa. The grey colour indicates the known optimum range of these predictors for malaria transmission. 5](#_Toc208569581)

[Scatter Plot of Malaria cases and Climatic Predictors 6](#_Toc208569582)

[**Figure C:** Relation between different meteorological variables and malaria cases in North Goa. 6](#_Toc208569583)

[**Figure D:** Relation between different meteorological variables and malaria cases in South Goa. 7](#_Toc208569584)

[Correlation Plot of Climatic Predictors 8](#_Toc208569585)

[**Figure E:** Correlation plot of meteorological variables to assess multi-collinearity between predictor variables. 8](#_Toc208569586)

[SHAP Analysis for Variable Selection 9](#_Toc208569587)

[**Figure F:** Plot of Shap variables indicating influence of different predictors on the model outputs. 9](#_Toc208569588)

[Process Flow Chart 10](#_Toc208569589)

[**Figure G:** Process flowchart detailing the steps involved in the modelling procedure 10](#_Toc208569590)

[Trends in predictor and outcome variables in North Goa and South Goa 11](#_Toc208569591)

[**Table A:** Results of seasonal Mann-Kendall test for assessing monotonous trends in North Goa and South Goa, India. 11](#_Toc208569592)

[Error Rates of Machine Learning Models in the Training dataset 12](#_Toc208569593)

[**Table B:** Error rates (RMSE & MAE) in the training dataset in North Goa and South Goa for the years 2018, and 2019 using three machine learning models 12](#_Toc208569594)

[Error Rates of Time Series Models in the Training dataset 12](#_Toc208569595)

[**Table C:** Error rates (RMSE & MAE) in the training dataset in North Goa and South Goa for the years 2018, and 2019 using three time series models. 12](#_Toc208569596)

[Error Rates of Hybrid ML-ARMA Models in the Training dataset 12](#_Toc208569597)

[**Table D:** Error rates (RMSE & MAE) in the training dataset in North Goa and South Goa for the years 2018, and 2019 using three machine learning models with time series features 12](#_Toc208569598)

[Point forecast values of malaria cases using Machine Learning Models 13](#_Toc208569599)

[**Table E:** Comparison of the forecasted cases of malaria and their 95% confidence intervals for Random Forest Model in North Goa and South Goa districts. 13](#_Toc208569600)

[**Table F:** Comparison of the forecasted cases of malaria and their 95% confidence intervals for SVM models in North Goa and South Goa districts. 14](#_Toc208569601)

[**Table G:** Comparison of the forecasted cases of malaria and their 95% confidence intervals for XGB models in North Goa and South Goa districts. 15](#_Toc208569602)

[Point forecast values of malaria cases using Time Series Models 16](#_Toc208569603)

[**Table H:** Comparison of the forecasted cases of malaria and their 95% confidence intervals for ARIMA models in North Goa and South Goa districts. 16](#_Toc208569604)

[**Table I:** Comparison of the forecasted cases of malaria and their 95% confidence intervals for SARIMA models in North Goa and South Goa districts. 17](#_Toc208569605)

[**Table J:** Comparison of the forecasted cases of malaria and their 95% confidence intervals for SARIMAX models in North Goa and South Goa districts. 18](#_Toc208569606)

[Point forecast values of malaria cases using Hybrid ML-ARMA Models 19](#_Toc208569607)

[**Table K:** Comparison of the forecasted cases of malaria and their 95% confidence intervals for RF model with time series features in North Goa and South Goa districts. 19](#_Toc208569608)

[**Table L:** Comparison of the forecasted cases of malaria and their 95% confidence intervals for SVM model with time series features in North Goa and South Goa districts. 20](#_Toc208569609)

[**Table M:** Comparison of the forecasted cases of malaria and their 95% confidence intervals for XGB model with time series features in North Goa and South Goa districts. 21](#_Toc208569610)

[**Table N:** Pair-wise Deibold-Mariano test for comparing the significance of differences between model forecasts 22](#_Toc208569611)

# Seasonality Plot of Malaria cases and Climatic Predictors


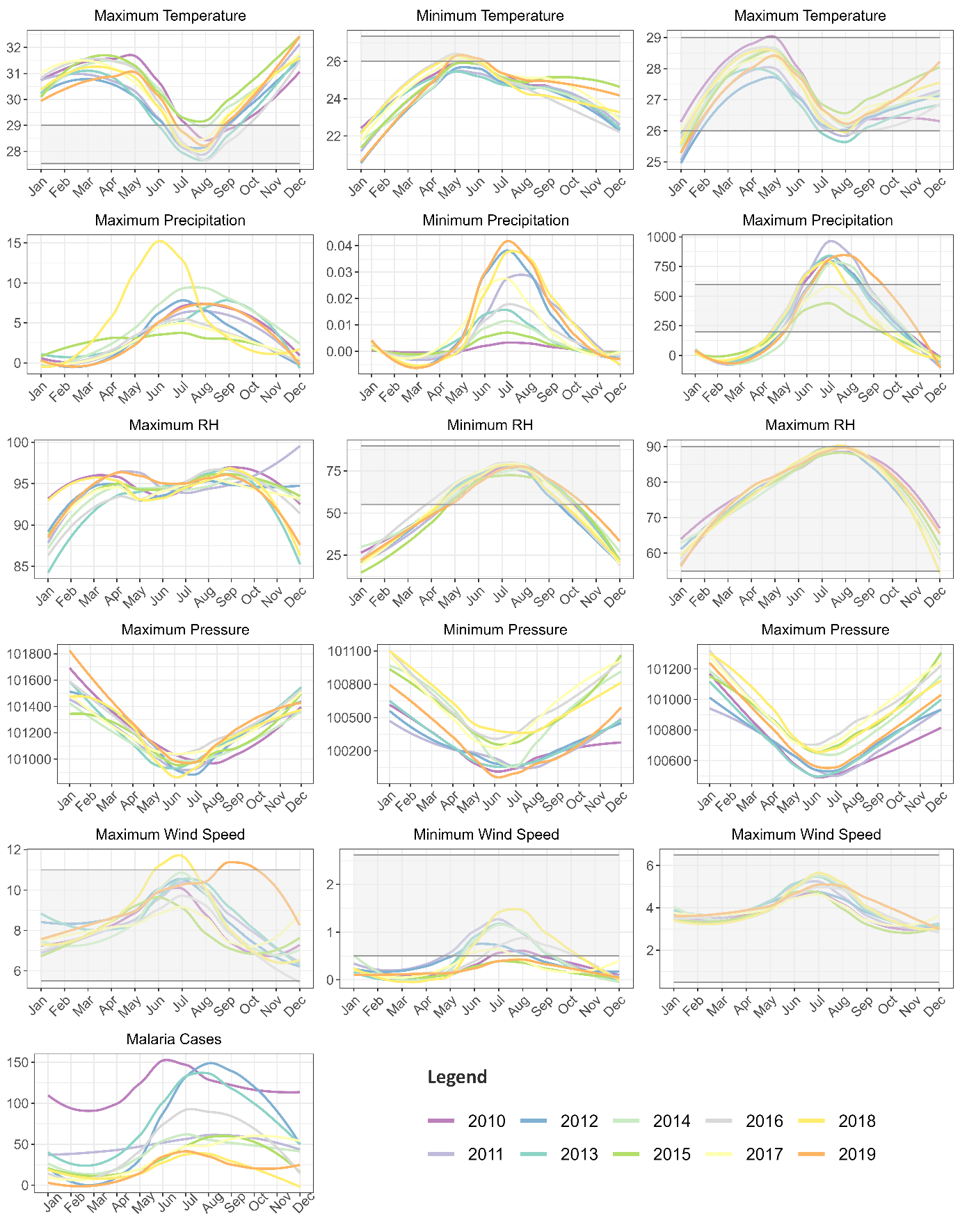


**Figure A:** Seasonal plots of all fifteen predictor variables and malaria cases in North Goa. The grey colour indicates the known optimum range of these predictors for malaria transmission.

**
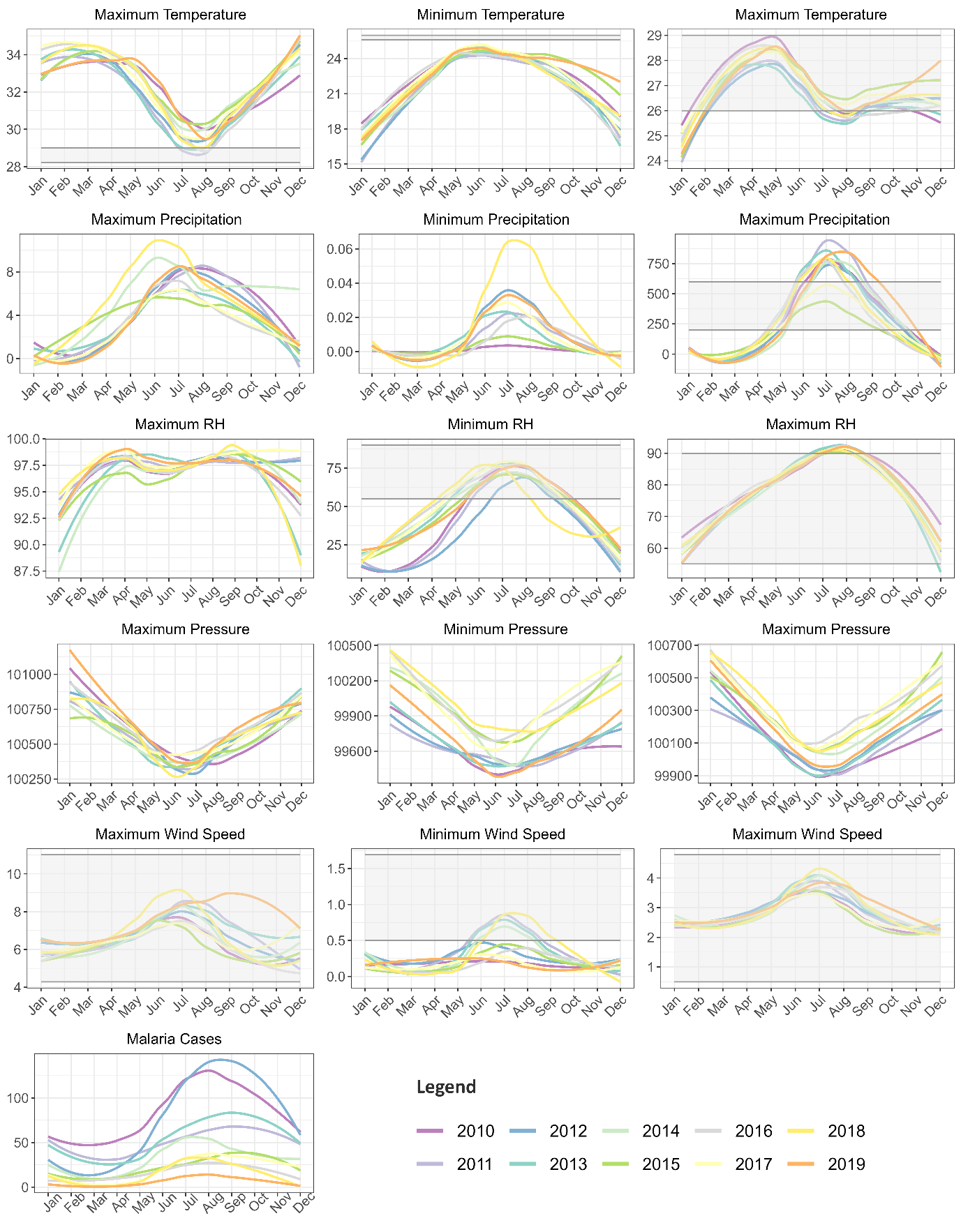
**

**Figure B:** Seasonal plots of all fifteen predictor variables and malaria cases in South Goa. The grey colour indicates the known optimum range of these predictors for malaria transmission.

# Scatter Plot of Malaria cases and Climatic Predictors


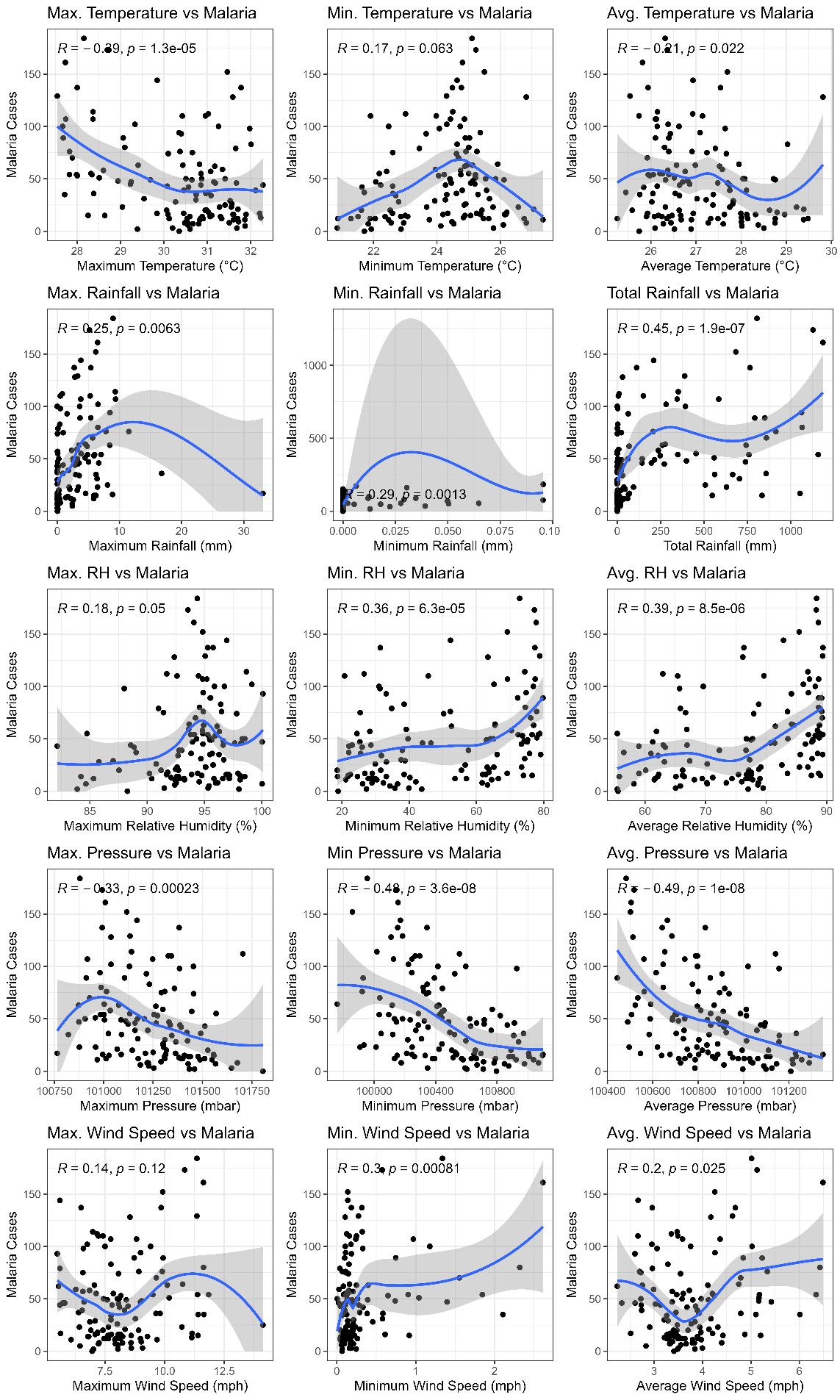


**Figure C:** Relation between different meteorological variables and malaria cases in North Goa.

**
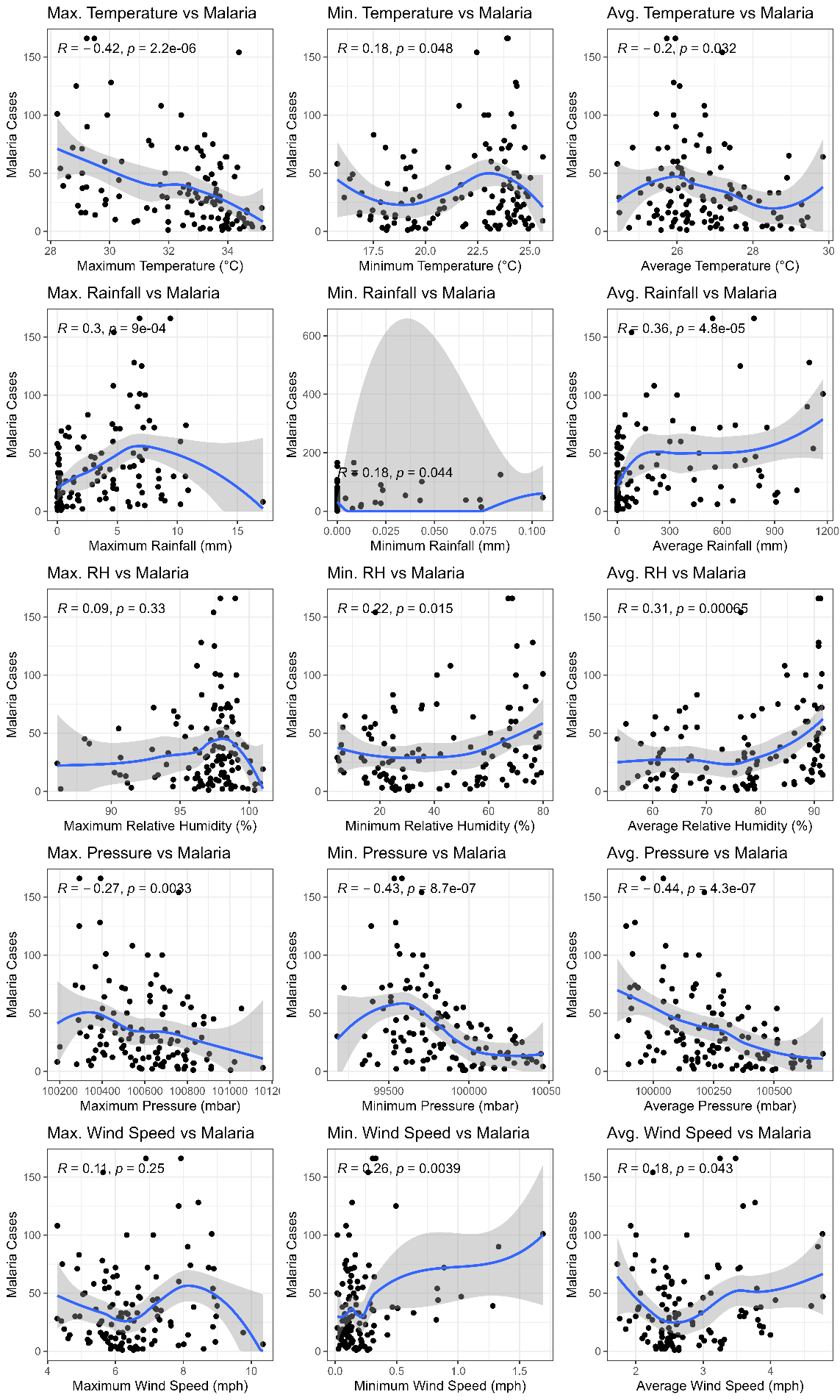
**

**Figure D:** Relation between different meteorological variables and malaria cases in South Goa.

# Correlation Plot of Climatic Predictors


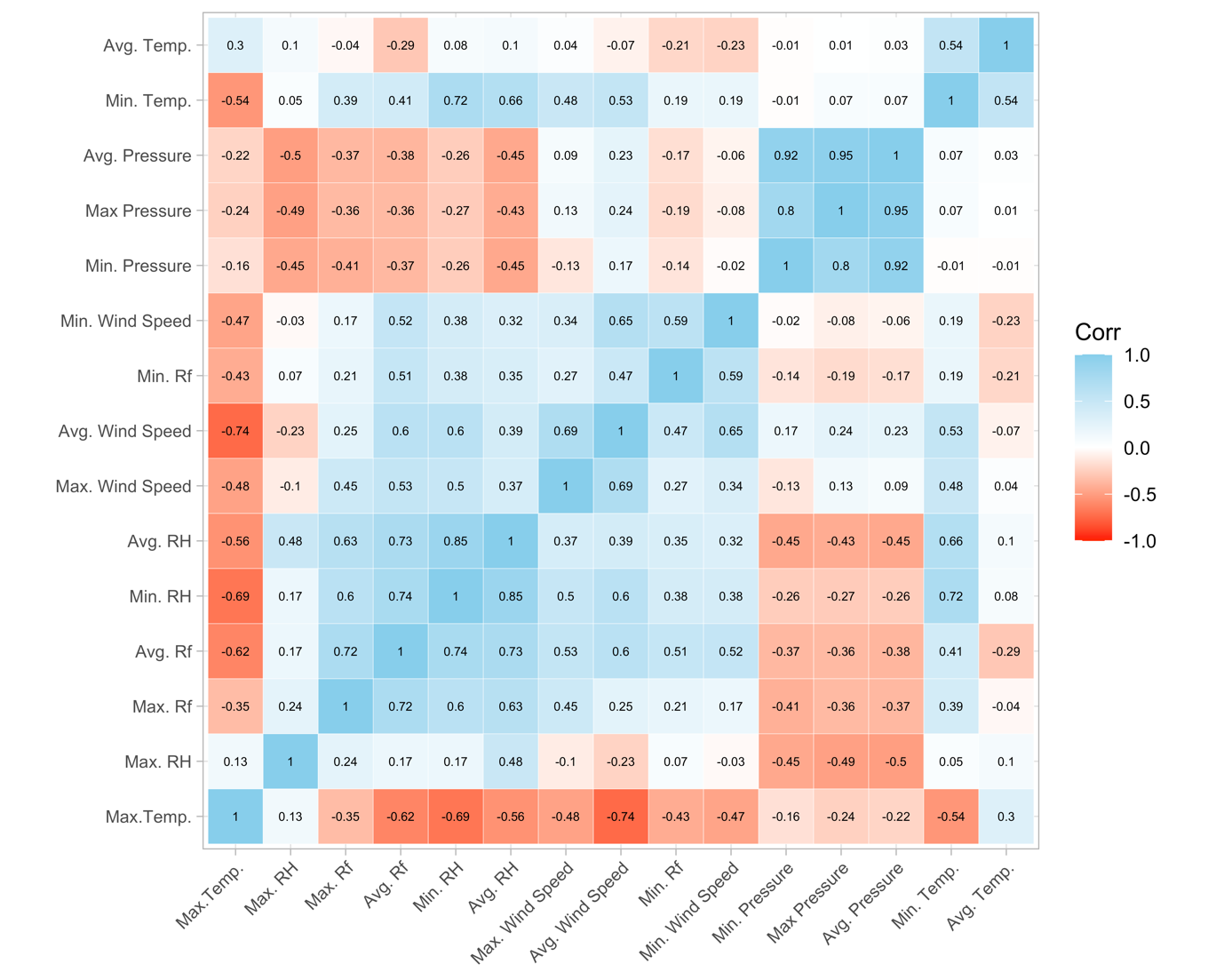


**Figure E:** Correlation plot of meteorological variables to assess multi-collinearity between predictor variables.

# SHAP Analysis for Variable Selection

**
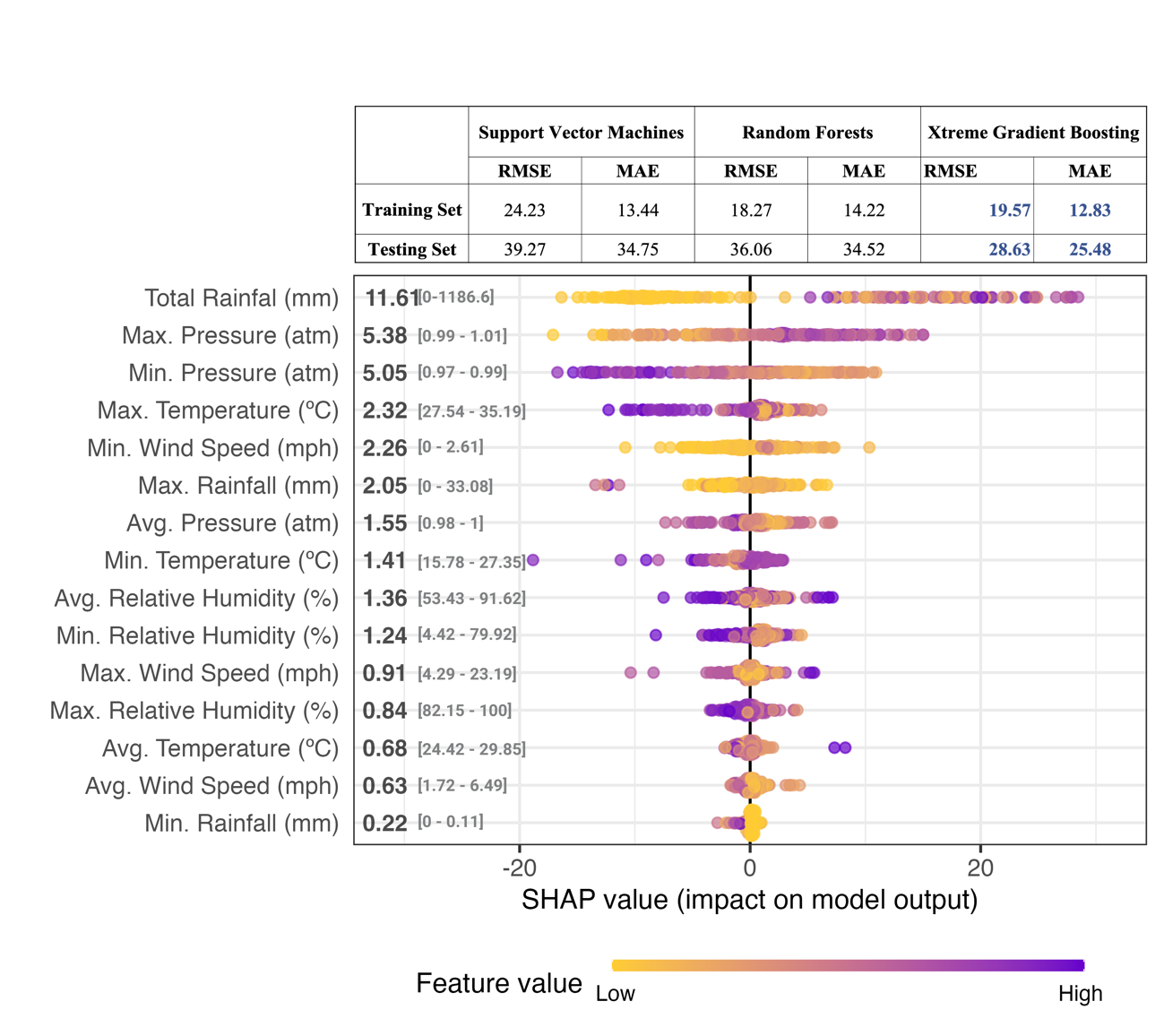
**

**Figure F:** Plot of Shap variables indicating influence of different predictors on the model outputs.

# Process Flow Chart

**
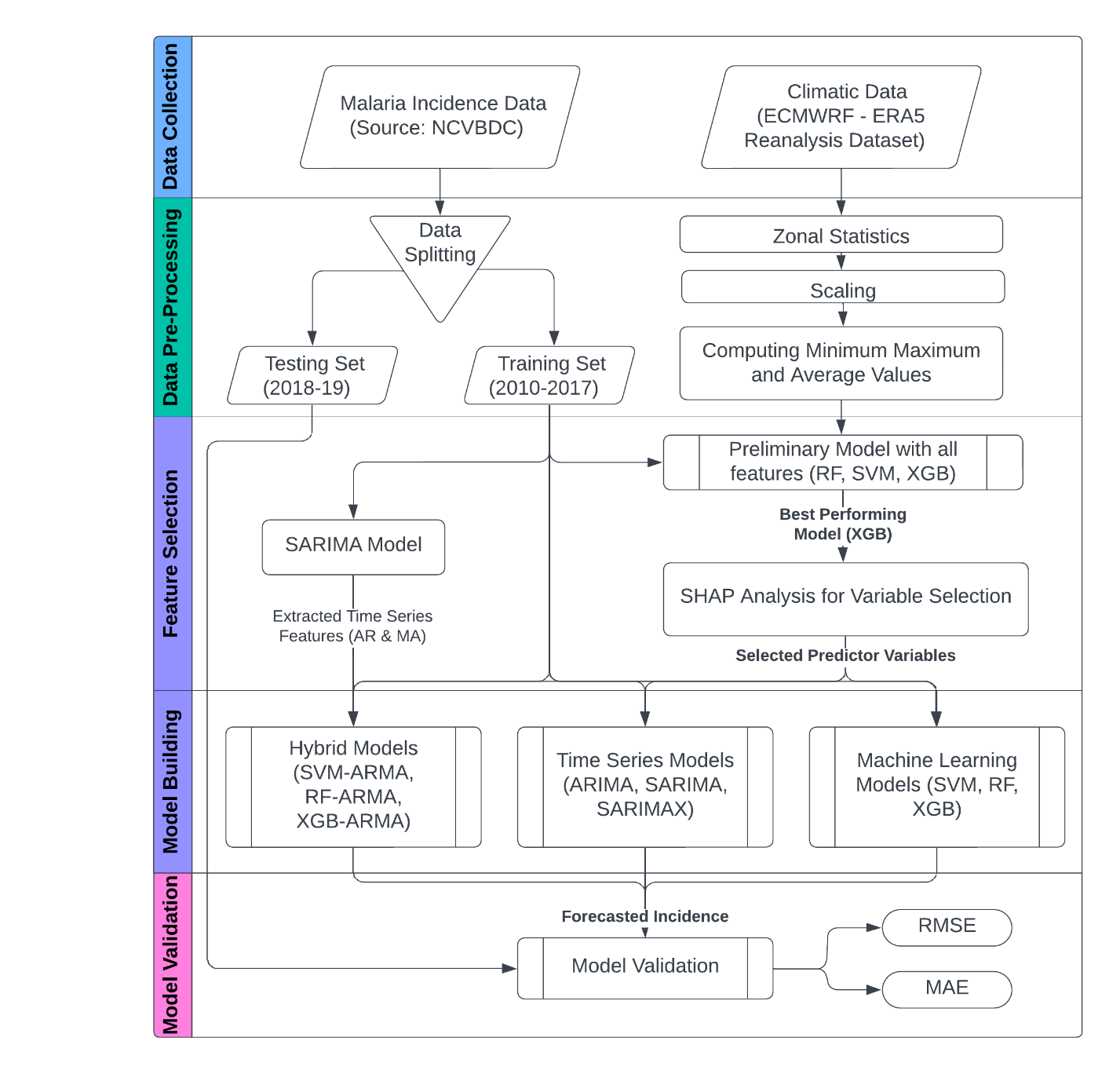
**

**Figure G:** Process flowchart detailing the steps involved in the modelling procedure

# Trends in predictor and outcome variables in North Goa and South Goa

**Table A:** Results of seasonal Mann-Kendall test for assessing monotonous trends in North Goa and South Goa, India.

|  | **Seasonal Mann-Kendall Test** | |
| --- | --- | --- |
|  | **North Goa** | **South Goa** |
| Max. Temperature | 1·68 *(P=0·09)* | 2·79 ***(P=0·01)*** |
| Min. Temperature | 3·31 ***(P=0·00)*** | 2·95 ***(P=0·00)*** |
| Avg. Temperature | 3·54 ***(P=0·00)*** | 3·9 ***(P=0·00)*** |
| Max. Rainfall | 1·56 *(P=0·12)* | 1·21 *(P=0·23)* |
| Min. Rainfall | -2·03 ***(P=0·04)*** | -2·25 ***(P=0·02)*** |
| Total Rainfall | 1·21 *(P=0·23)* | 1·09 *(P=0·28)* |
| Max. Relative Humidity | 0·38 *(P=0·71)* | 1·96 ***(P=0·05)*** |
| Min. Relative Humidity | 1·96 ***(P=0·05)*** | 2 ***(P=0·05)*** |
| Avg. Relative Humidity | 1·37 *(P=0·17)* | 0·73 *(P=0·46)* |
| Max. Pressure | 2·24 ***(P=0·03)*** | 2·36 ***(P=0·02)*** |
| Min. Pressure | 0·77 *(P=0·44)* | 0·97 *(P=0·33)* |
| Avg. Pressure | 2·51 ***(P=0·01)*** | 2·83 ***(P=0·00)*** |
| Max. Wind Speed | 1·68 *(P=0·09)* | 2·2 ***(P=0·03)*** |
| Min. Wind Speed | -2·47 ***(P=0·01)*** | -2·71 ***(P=0·01)*** |
| Avg. Wind Speed | -0·1 *(P=0·92)* | -0·49 *(P=0·62)* |
| Malaria Cases | -10·86 ***(P<0·01)*** | -12·92 ***(P<0·01)*** |

# Error Rates of Machine Learning Models in the Training dataset

**Table B:** Error rates (RMSE & MAE) in the training dataset in North Goa and South Goa for the years 2018, and 2019 using three machine learning models

|  | **Forecasted Year** | **SVM** | | **RF** | | **XGB** | |
| --- | --- | --- | --- | --- | --- | --- | --- |
|  |  | **RMSE** | **MAE** | **RMSE** | **MAE** | **RMSE** | **MAE** |
| **North Goa** | 2018 | 26·83 | 18·41 | 14·08 | 10·24 | 26·15 | 17·23 |
|  | 2019 | 27·36 | 18·47 | 14·83 | 10·60 | 25·82 | 17·00 |
| **South Goa** | 2018 | 24·02 | 12·96 | 14·71 | 9·79 | 24·83 | 14·73 |
|  | 2019 | 23·29 | 12·66 | 14·06 | 9·19 | 23·58 | 13·89 |

# Error Rates of Time Series Models in the Training dataset

**Table C:** Error rates (RMSE & MAE) in the training dataset in North Goa and South Goa for the years 2018, and 2019 using three time series models.

|  | **Forecasted Year** | **ARIMA** | | **SARIMA** | | **SARIMAX** | |
| --- | --- | --- | --- | --- | --- | --- | --- |
|  |  | **RMSE** | **MAE** | **RMSE** | **MAE** | **RMSE** | **MAE** |
| **North Goa** | 2018 | 25·73 | 16·51 | 20·53 | 12·79 | 19·23 | 12·71 |
|  | 2019 | 26·12 | 16·99 | 20·97 | 13·73 | 17·03 | 11·76 |
| **South Goa** | 2018 | 21·45 | 14·95 | 16·50 | 10·39 | 14·30 | 9·06 |
|  | 2019 | 20·65 | 14·50 | 16·21 | 10·06 | 14·29 | 8·79 |

# Error Rates of Hybrid ML-ARMA Models in the Training dataset

**Table D:** Error rates (RMSE & MAE) in the training dataset in North Goa and South Goa for the years 2018, and 2019 using three machine learning models with time series features

|  | **Forecasted Year** | **SVM-ARMA** | | **RF-ARMA** | | **XGB-ARMA** | |
| --- | --- | --- | --- | --- | --- | --- | --- |
|  |  | **RMSE** | **MAE** | **RMSE** | **MAE** | **RMSE** | **MAE** |
| **North Goa** | 2018 | 1·15 | 0·85 | 6·39 | 4·07 | 1·98 | 0·85 |
|  | 2019 | 1·12 | 0·79 | 6·09 | 3·80 | 0·94 | 0·32 |
| **South Goa** | 2018 | 18·57 | 11·33 | 11·63 | 7·39 | 13·42 | 7·70 |
|  | 2019 | 10·39 | 7·39 | 10·01 | 6·15 | 3·66 | 1·60 |

# Point forecast values of malaria cases using Machine Learning Models

**Table E:** Comparison of the forecasted cases of malaria and their 95% confidence intervals for Random Forest Model in North Goa and South Goa districts.

|  | **NORTH GOA** | | | **SOUTH GOA** | | |
| --- | --- | --- | --- | --- | --- | --- |
| **Month-Year** | **Malaria Cases** | **Forecasts** | **95% CI** | **Malaria Cases** | **Forecasts** | **95% CI** |
| Jan-18 | 17 | 21 | [16, 25] | 12 | 23 | [18, 27] |
| Feb-18 | 15 | 22 | [18, 27] | 4 | 22 | [14, 29] |
| Mar-18 | 7 | 35 | [29, 41] | 2 | 26 | [20, 32] |
| Apr-18 | 12 | 22 | [18, 26] | 5 | 16 | [13, 20] |
| May-18 | 11 | 22 | [18, 26] | 5 | 16 | [13, 19] |
| Jun-18 | 17 | 74 | [66, 83] | 8 | 38 | [31, 45] |
| Jul-18 | 54 | 95 | [82, 107] | 47 | 57 | [46, 68] |
| Aug-18 | 35 | 101 | [91, 111] | 39 | 68 | [56, 80] |
| Sep-18 | 23 | 73 | [65, 81] | 8 | 56 | [47, 66] |
| Oct-18 | 17 | 55 | [47, 64] | 19 | 26 | [19, 33] |
| Nov-18 | 5 | 40 | [32, 48] | 11 | 20 | [15, 25] |
| Dec-18 | 2 | 27 | [21, 32] | 2 | 25 | [20, 31] |
| Jan-19 | 0 | 22 | [17, 27] | 3 | 24 | [18, 29] |
| Feb-19 | 3 | 25 | [20, 30] | 1 | 22 | [17, 28] |
| Mar-19 | 2 | 25 | [20, 30] | 1 | 34 | [26, 42] |
| Apr-19 | 3 | 27 | [20, 34] | 2 | 34 | [25, 43] |
| May-19 | 8 | 29 | [23, 36] | 2 | 37 | [28, 46] |
| Jun-19 | 23 | 60 | [50, 71] | 6 | 32 | [24, 40] |
| Jul-19 | 76 | 88 | [74, 101] | 14 | 79 | [66, 93] |
| Aug-19 | 15 | 81 | [71, 91] | 16 | 72 | [60, 84] |
| Sep-19 | 15 | 63 | [56, 69] | 10 | 42 | [35, 49] |
| Oct-19 | 25 | 65 | [56, 75] | 6 | 43 | [35, 51] |
| Nov-19 | 13 | 33 | [26, 41] | 2 | 32 | [22, 41] |
| Dec-19 | 29 | 25 | [19, 32] | 4 | 22 | [16, 28] |

**Table F:** Comparison of the forecasted cases of malaria and their 95% confidence intervals for SVM models in North Goa and South Goa districts.

|  | **NORTH GOA** | | | **SOUTH GOA** | | |
| --- | --- | --- | --- | --- | --- | --- |
| **Month-Year** | **Malaria Cases** | **Forecasts** | **95% CI** | **Malaria Cases** | **Forecasts** | **95% CI** |
| Jan-18 | 17 | 20 | [17, 24] | 12 | 14 | [12, 17] |
| Feb-18 | 15 | 17 | [13, 21] | 4 | 7 | [4, 10] |
| Mar-18 | 7 | 24 | [20, 29] | 2 | 17 | [13, 21] |
| Apr-18 | 12 | 27 | [23, 31] | 5 | 14 | [10, 17] |
| May-18 | 11 | 17 | [12, 22] | 5 | 14 | [10, 18] |
| Jun-18 | 17 | 65 | [54, 76] | 8 | 23 | [11, 35] |
| Jul-18 | 54 | 76 | [66, 85] | 47 | 51 | [40, 63] |
| Aug-18 | 35 | 76 | [66, 85] | 39 | 50 | [37, 63] |
| Sep-18 | 23 | 69 | [59, 78] | 8 | 58 | [50, 65] |
| Oct-18 | 17 | 37 | [30, 43] | 19 | 24 | [20, 28] |
| Nov-18 | 5 | 27 | [21, 32] | 11 | 14 | [12, 16] |
| Dec-18 | 2 | 38 | [34, 42] | 2 | 35 | [29, 40] |
| Jan-19 | 0 | 25 | [22, 29] | 3 | 30 | [26, 33] |
| Feb-19 | 3 | 24 | [19, 30] | 1 | 36 | [32, 40] |
| Mar-19 | 2 | 32 | [25, 39] | 1 | 40 | [34, 46] |
| Apr-19 | 3 | 29 | [25, 33] | 2 | 27 | [20, 33] |
| May-19 | 8 | 27 | [22, 32] | 2 | 29 | [23, 34] |
| Jun-19 | 23 | 52 | [43, 61] | 6 | 22 | [11, 33] |
| Jul-19 | 76 | 74 | [61, 86] | 14 | 49 | [34, 64] |
| Aug-19 | 15 | 68 | [60, 76] | 16 | 42 | [30, 55] |
| Sep-19 | 15 | 70 | [61, 79] | 10 | 48 | [40, 55] |
| Oct-19 | 25 | 58 | [50, 66] | 6 | 37 | [28, 46] |
| Nov-19 | 13 | 30 | [25, 34] | 2 | 27 | [22, 32] |
| Dec-19 | 29 | 29 | [25, 34] | 4 | 38 | [33, 43] |

**Table G:** Comparison of the forecasted cases of malaria and their 95% confidence intervals for XGB models in North Goa and South Goa districts.

|  | **NORTH GOA** | | | **SOUTH GOA** | | |
| --- | --- | --- | --- | --- | --- | --- |
| **Month-Year** | **Malaria Cases** | **Forecasts** | **95% CI** | **Malaria Cases** | **Forecasts** | **95% CI** |
| Jan-18 | 17 | 16 | [15, 17] | 12 | 16 | [15, 17] |
| Feb-18 | 15 | 19 | [18, 21] | 4 | 11 | [10, 12] |
| Mar-18 | 7 | 22 | [21, 23] | 2 | 16 | [15, 17] |
| Apr-18 | 12 | 14 | [13, 15] | 5 | 12 | [11, 13] |
| May-18 | 11 | 19 | [18, 20] | 5 | 13 | [12, 14] |
| Jun-18 | 17 | 49 | [47, 50] | 8 | 40 | [38, 42] |
| Jul-18 | 54 | 66 | [64, 68] | 47 | 32 | [30, 33] |
| Aug-18 | 35 | 68 | [67, 70] | 39 | 31 | [30, 33] |
| Sep-18 | 23 | 56 | [54, 57] | 8 | 53 | [51, 54] |
| Oct-18 | 17 | 44 | [43, 46] | 19 | 18 | [17, 19] |
| Nov-18 | 5 | 31 | [29, 32] | 11 | 12 | [11, 13] |
| Dec-18 | 2 | 21 | [20, 23] | 2 | 18 | [17, 19] |
| Jan-19 | 0 | 28 | [27, 29] | 3 | 31 | [30, 32] |
| Feb-19 | 3 | 31 | [30, 32] | 1 | 18 | [16, 20] |
| Mar-19 | 2 | 28 | [27, 29] | 1 | 31 | [30, 33] |
| Apr-19 | 3 | 28 | [27, 29] | 2 | 19 | [18, 21] |
| May-19 | 8 | 38 | [38, 39] | 2 | 28 | [26, 30] |
| Jun-19 | 23 | 64 | [63, 65] | 6 | 30 | [28, 32] |
| Jul-19 | 76 | 82 | [81, 84] | 14 | 41 | [38, 43] |
| Aug-19 | 15 | 80 | [78, 81] | 16 | 60 | [57, 62] |
| Sep-19 | 15 | 66 | [65, 67] | 10 | 45 | [42, 47] |
| Oct-19 | 25 | 66 | [65, 67] | 6 | 47 | [44, 49] |
| Nov-19 | 13 | 35 | [34, 36] | 2 | 17 | [15, 18] |
| Dec-19 | 29 | 29 | [28, 30] | 4 | 17 | [16, 19] |

# Point forecast values of malaria cases using Time Series Models

**Table H:** Comparison of the forecasted cases of malaria and their 95% confidence intervals for ARIMA models in North Goa and South Goa districts.

|  | **NORTH GOA** | | | **SOUTH GOA** | | |
| --- | --- | --- | --- | --- | --- | --- |
| **Month-Year** | **Malaria Cases** | **Forecasts** | **95% CI** | **Malaria Cases** | **Forecasts** | **95% CI** |
| Jan-18 | 17 | 17 | [5, 46] | 12 | 22 | [7, 56] |
| Feb-18 | 15 | 9 | [1, 37] | 4 | 18 | [4, 57] |
| Mar-18 | 7 | 6 | [0, 34] | 2 | 16 | [3, 61] |
| Apr-18 | 12 | 7 | [0, 38] | 5 | 15 | [2, 65] |
| May-18 | 11 | 10 | [0, 53] | 5 | 14 | [2, 65] |
| Jun-18 | 17 | 18 | [2, 84] | 8 | 15 | [2, 69] |
| Jul-18 | 54 | 33 | [5, 132] | 47 | 17 | [2, 73] |
| Aug-18 | 35 | 52 | [9, 189] | 39 | 18 | [2, 78] |
| Sep-18 | 23 | 67 | [13, 233] | 8 | 19 | [3, 82] |
| Oct-18 | 17 | 69 | [13, 246] | 19 | 19 | [3, 84] |
| Nov-18 | 5 | 57 | [9, 225] | 11 | 19 | [3, 84] |
| Dec-18 | 2 | 39 | [4, 186] | 2 | 19 | [3, 84] |
| Jan-19 | 0 | 3 | [0, 11] | 3 | 5 | [0, 20] |
| Feb-19 | 3 | 4 | [0, 22] | 1 | 5 | [0, 26] |
| Mar-19 | 2 | 8 | [0, 46] | 1 | 6 | [0, 32] |
| Apr-19 | 3 | 16 | [1, 88] | 2 | 12 | [0, 57] |
| May-19 | 8 | 24 | [2, 123] | 2 | 13 | [0, 62] |
| Jun-19 | 23 | 30 | [3, 144] | 6 | 15 | [1, 67] |
| Jul-19 | 76 | 29 | [3, 142] | 14 | 17 | [1, 72] |
| Aug-19 | 15 | 24 | [2, 123] | 16 | 14 | [0, 65] |
| Sep-19 | 15 | 18 | [1, 100] | 10 | 12 | [0, 60] |
| Oct-19 | 25 | 14 | [1, 81] | 6 | 11 | [0, 55] |
| Nov-19 | 13 | 11 | [0, 69] | 2 | 9 | [0, 50] |
| Dec-19 | 29 | 10 | [0, 66] | 4 | 9 | [0, 50] |

**Table I:** Comparison of the forecasted cases of malaria and their 95% confidence intervals for SARIMA models in North Goa and South Goa districts.

|  | **NORTH GOA** | | | **SOUTH GOA** | | |
| --- | --- | --- | --- | --- | --- | --- |
| **Month-Year** | **Malaria Cases** | **Forecasts** | **95% CI** | **Malaria Cases** | **Forecasts** | **95% CI** |
| Jan-18 | 17 | 19 | [7, 46] | 12 | 10 | [3, 26] |
| Feb-18 | 15 | 13 | [3, 37] | 4 | 7 | [2, 20] |
| Mar-18 | 7 | 10 | [2, 33] | 2 | 3 | [0, 10] |
| Apr-18 | 12 | 13 | [2, 41] | 5 | 5 | [1, 14] |
| May-18 | 11 | 16 | [3, 52] | 5 | 11 | [3, 29] |
| Jun-18 | 17 | 38 | [10, 106] | 8 | 12 | [3, 32] |
| Jul-18 | 54 | 53 | [16, 141] | 47 | 21 | [7, 53] |
| Aug-18 | 35 | 46 | [13, 127] | 39 | 25 | [9, 63] |
| Sep-18 | 23 | 38 | [10, 107] | 8 | 19 | [6, 49] |
| Oct-18 | 17 | 41 | [11, 115] | 19 | 22 | [7, 57] |
| Nov-18 | 5 | 37 | [10, 107] | 11 | 17 | [5, 45] |
| Dec-18 | 2 | 17 | [3, 57] | 2 | 10 | [3, 29] |
| Jan-19 | 0 | 1 | [0, 6] | 3 | 3 | [0, 13] |
| Feb-19 | 3 | 2 | [0, 10] | 1 | 2 | [0, 8] |
| Mar-19 | 2 | 2 | [0, 11] | 1 | 1 | [0, 5] |
| Apr-19 | 3 | 3 | [0, 21] | 2 | 2 | [0, 10] |
| May-19 | 8 | 6 | [0, 35] | 2 | 3 | [0, 13] |
| Jun-19 | 23 | 17 | [1, 81] | 6 | 4 | [0, 17] |
| Jul-19 | 76 | 35 | [5, 150] | 14 | 20 | [5, 53] |
| Aug-19 | 15 | 33 | [4, 144] | 16 | 24 | [7, 61] |
| Sep-19 | 15 | 27 | [3, 128] | 10 | 6 | [0, 23] |
| Oct-19 | 25 | 29 | [3, 137] | 6 | 13 | [2, 38] |
| Nov-19 | 13 | 23 | [2, 115] | 2 | 8 | [1, 28] |
| Dec-19 | 29 | 10 | [0, 64] | 4 | 4 | [0, 16] |

**Table J:** Comparison of the forecasted cases of malaria and their 95% confidence intervals for SARIMAX models in North Goa and South Goa districts.

|  | **NORTH GOA** | | | **SOUTH GOA** | | |
| --- | --- | --- | --- | --- | --- | --- |
| **Month-Year** | **Malaria Cases** | **Forecasts** | **95% CI** | **Malaria Cases** | **Forecasts** | **95% CI** |
| Jan-18 | 17 | 18 | [6, 43] | 12 | 26 | [12, 53] |
| Feb-18 | 15 | 13 | [3, 40] | 4 | 22 | [9, 50] |
| Mar-18 | 7 | 7 | [1, 27] | 2 | 12 | [3, 34] |
| Apr-18 | 12 | 11 | [1, 40] | 5 | 19 | [5, 56] |
| May-18 | 11 | 16 | [3, 56] | 5 | 34 | [9, 100] |
| Jun-18 | 17 | 41 | [10, 121] | 8 | 38 | [10, 118] |
| Jul-18 | 54 | 53 | [14, 151] | 47 | 59 | [15, 183] |
| Aug-18 | 35 | 46 | [12, 136] | 39 | 53 | [13, 177] |
| Sep-18 | 23 | 33 | [7, 102] | 8 | 38 | [8, 138] |
| Oct-18 | 17 | 54 | [14, 154] | 19 | 65 | [14, 231] |
| Nov-18 | 5 | 45 | [11, 133] | 11 | 59 | [12, 220] |
| Dec-18 | 2 | 17 | [3, 61] | 2 | 44 | [8, 179] |
| Jan-19 | 0 | 1 | [0, 4] | 3 | 5 | [1, 17] |
| Feb-19 | 3 | 1 | [0, 5] | 1 | 5 | [0, 17] |
| Mar-19 | 2 | 1 | [0, 5] | 1 | 1 | [0, 7] |
| Apr-19 | 3 | 3 | [0, 15] | 2 | 6 | [0, 20] |
| May-19 | 8 | 3 | [0, 17] | 2 | 6 | [0, 20] |
| Jun-19 | 23 | 15 | [2, 54] | 6 | 16 | [3, 43] |
| Jul-19 | 76 | 19 | [3, 65] | 14 | 22 | [6, 56] |
| Aug-19 | 15 | 18 | [3, 63] | 16 | 27 | [8, 67] |
| Sep-19 | 15 | 12 | [1, 45] | 10 | 9 | [1, 30] |
| Oct-19 | 25 | 12 | [2, 47] | 6 | 27 | [7, 67] |
| Nov-19 | 13 | 12 | [2, 47] | 2 | 20 | [5, 53] |
| Dec-19 | 29 | 6 | [0, 27] | 4 | 15 | [3, 44] |

# Point forecast values of malaria cases using Hybrid ML-ARMA Models

**Table K:** Comparison of the forecasted cases of malaria and their 95% confidence intervals for RF model with time series features in North Goa and South Goa districts.

|  | **NORTH GOA** | | | **SOUTH GOA** | | |
| --- | --- | --- | --- | --- | --- | --- |
| **Month-Year** | **Malaria Cases** | **Forecasts** | **95% CI** | **Malaria Cases** | **Forecasts** | **95% CI** |
| Jan-18 | 17 | 14 | [9, 20] | 12 | 17 | [16, 19] |
| Feb-18 | 15 | 13 | [11, 16] | 4 | 13 | [12, 14] |
| Mar-18 | 7 | 9 | [5, 12] | 2 | 14 | [13, 16] |
| Apr-18 | 12 | 10 | [8, 12] | 5 | 11 | [10, 12] |
| May-18 | 11 | 14 | [10, 18] | 5 | 12 | [10, 14] |
| Jun-18 | 17 | 22 | [13, 30] | 8 | 22 | [20, 23] |
| Jul-18 | 54 | 45 | [34, 57] | 47 | 38 | [35, 41] |
| Aug-18 | 35 | 49 | [39, 59] | 39 | 51 | [46, 55] |
| Sep-18 | 23 | 24 | [17, 30] | 8 | 31 | [29, 34] |
| Oct-18 | 17 | 20 | [13, 27] | 19 | 21 | [19, 23] |
| Nov-18 | 5 | 20 | [13, 27] | 11 | 16 | [14, 18] |
| Dec-18 | 2 | 11 | [8, 14] | 2 | 15 | [14, 17] |
| Jan-19 | 0 | 7 | [2, 13] | 3 | 9 | [8, 11] |
| Feb-19 | 3 | 8 | [0, 17] | 1 | 7 | [6, 8] |
| Mar-19 | 2 | 9 | [4, 15] | 1 | 10 | [8, 11] |
| Apr-19 | 3 | 11 | [7, 15] | 2 | 10 | [9, 11] |
| May-19 | 8 | 10 | [5, 15] | 2 | 13 | [12, 14] |
| Jun-19 | 23 | 23 | [15, 30] | 6 | 13 | [12, 15] |
| Jul-19 | 76 | 66 | [56, 77] | 14 | 46 | [42, 49] |
| Aug-19 | 15 | 47 | [39, 56] | 16 | 47 | [44, 51] |
| Sep-19 | 15 | 22 | [12, 32] | 10 | 17 | [15, 19] |
| Oct-19 | 25 | 23 | [16, 30] | 6 | 22 | [20, 24] |
| Nov-19 | 13 | 17 | [8, 26] | 2 | 13 | [12, 15] |
| Dec-19 | 29 | 16 | [10, 23] | 4 | 9 | [8, 10] |

**Table L:** Comparison of the forecasted cases of malaria and their 95% confidence intervals for SVM model with time series features in North Goa and South Goa districts.

|  | **NORTH GOA** | | | **SOUTH GOA** | | |
| --- | --- | --- | --- | --- | --- | --- |
| **Month-Year** | **Malaria Cases** | **Forecasts** | **95% CI** | **Malaria Cases** | **Forecasts** | **95% CI** |
| Jan-18 | 17 | 16 | [10, 21] | 12 | 11 | [9, 12] |
| Feb-18 | 15 | 14 | [11, 17] | 4 | 7 | [5, 9] |
| Mar-18 | 7 | 7 | [4, 10] | 2 | 6 | [4, 7] |
| Apr-18 | 12 | 12 | [9, 15] | 5 | 8 | [7, 10] |
| May-18 | 11 | 12 | [7, 17] | 5 | 9 | [5, 13] |
| Jun-18 | 17 | 17 | [11, 23] | 8 | 20 | [18, 23] |
| Jul-18 | 54 | 54 | [48, 61] | 47 | 39 | [35, 42] |
| Aug-18 | 35 | 36 | [32, 40] | 39 | 52 | [46, 57] |
| Sep-18 | 23 | 23 | [20, 26] | 8 | 30 | [23, 36] |
| Oct-18 | 17 | 17 | [14, 20] | 19 | 17 | [14, 19] |
| Nov-18 | 5 | 6 | [2, 9] | 11 | 11 | [4, 17] |
| Dec-18 | 2 | 2 | [0, 5] | 2 | 9 | [4, 13] |
| Jan-19 | 0 | 1 | [0, 4] | 3 | 8 | [5, 10] |
| Feb-19 | 3 | 2 | [0, 5] | 1 | 1 | [0, 4] |
| Mar-19 | 2 | 1 | [0, 6] | 1 | 3 | [1, 5] |
| Apr-19 | 3 | 3 | [0, 6] | 2 | 2 | [0, 5] |
| May-19 | 8 | 8 | [4, 13] | 2 | 6 | [0, 15] |
| Jun-19 | 23 | 23 | [17, 30] | 6 | 3 | [0, 8] |
| Jul-19 | 76 | 74 | [65, 83] | 14 | 23 | [20, 26] |
| Aug-19 | 15 | 18 | [10, 26] | 16 | 24 | [21, 27] |
| Sep-19 | 15 | 15 | [5, 24] | 10 | 21 | [18, 25] |
| Oct-19 | 25 | 25 | [20, 29] | 6 | 20 | [17, 22] |
| Nov-19 | 13 | 13 | [10, 17] | 2 | 7 | [3, 10] |
| Dec-19 | 29 | 28 | [24, 33] | 4 | 4 | [0, 9] |

**Table M:** Comparison of the forecasted cases of malaria and their 95% confidence intervals for XGB model with time series features in North Goa and South Goa districts.

|  | **NORTH GOA** | | | **SOUTH GOA** | | |
| --- | --- | --- | --- | --- | --- | --- |
| **Month-Year** | **Malaria Cases** | **Forecasts** | **95% CI** | **Malaria Cases** | **Forecasts** | **95% CI** |
| Jan-18 | 17 | 14 | [13, 16] | 12 | 15 | [14, 16] |
| Feb-18 | 15 | 13 | [12, 15] | 4 | 7 | [6, 8] |
| Mar-18 | 7 | 8 | [7, 9] | 2 | 12 | [11, 13] |
| Apr-18 | 12 | 9 | [8, 9] | 5 | 1 | [0, 2] |
| May-18 | 11 | 13 | [12, 14] | 5 | 6 | [4, 7] |
| Jun-18 | 17 | 23 | [22, 24] | 8 | 18 | [17, 20] |
| Jul-18 | 54 | 45 | [43, 48] | 47 | 32 | [30, 34] |
| Aug-18 | 35 | 39 | [37, 41] | 39 | 35 | [32, 38] |
| Sep-18 | 23 | 22 | [21, 24] | 8 | 27 | [25, 29] |
| Oct-18 | 17 | 18 | [16, 19] | 19 | 18 | [17, 20] |
| Nov-18 | 5 | 19 | [18, 21] | 11 | 22 | [20, 23] |
| Dec-18 | 2 | 9 | [8, 10] | 2 | 1 | [-1, 2] |
| Jan-19 | 0 | 5 | [4, 6] | 3 | 4 | [3, 6] |
| Feb-19 | 3 | 6 | [5, 7] | 1 | 3 | [2, 4] |
| Mar-19 | 2 | 6 | [5, 7] | 1 | 6 | [4, 7] |
| Apr-19 | 3 | 6 | [5, 6] | 2 | 1 | [0, 2] |
| May-19 | 8 | 8 | [7, 9] | 2 | 9 | [8, 10] |
| Jun-19 | 23 | 32 | [31, 33] | 6 | 7 | [6, 9] |
| Jul-19 | 76 | 78 | [76, 80] | 14 | 19 | [15, 22] |
| Aug-19 | 15 | 13 | [11, 15] | 16 | 16 | [13, 20] |
| Sep-19 | 15 | 15 | [14, 16] | 10 | 8 | [6, 10] |
| Oct-19 | 25 | 27 | [26, 28] | 6 | 6 | [4, 8] |
| Nov-19 | 13 | 11 | [10, 13] | 2 | 5 | [4, 7] |
| Dec-19 | 29 | 29 | [28, 30] | 4 | 3 | [2, 4] |

**Table N:** Pair-wise Deibold-Mariano test for comparing the significance of differences between model forecasts

|  |  | **North Goa** | | **South Goa** | |
| --- | --- | --- | --- | --- | --- |
| **Model 1** | **Model 2** | **Value** | **p-value** | **Value** | **p-value** |
| RF | SVM | 2.67 | 0.01 | 1.49 | 0.15 |
| XGB | RF | 2.04 | 0.05 | 2.28 | 0.03 |
| SVM | XGB | 0.17 | 0.87 | 0.81 | 0.42 |
| ARIMA | SARIMA | 2.30 | 0.03 | 3.71 | 0.00 |
| SARIMA | SARIMAX | 2.72 | 0.01 | -2.06 | 0.05 |
| SARIMAX | ARIMA | 2.54 | 0.02 | 0.72 | 0.48 |
| RF-ARMA | SVM-ARMA | 2.15 | 0.04 | 2.27 | 0.03 |
| SVM-ARMA | XGB-ARMA | 2.52 | 0.02 | 1.10 | 0.28 |
| XGB-ARMA | RF-ARMA | 1.59 | 0.13 | -2.42 | 0.02 |
| RF-ARMA | RF | -4.56 | 0.00 | -4.73 | 0.00 |
| RF-ARMA | XGB | -4.32 | 0.00 | -3.69 | 0.00 |
| RF-ARMA | SVM | -4.47 | 0.00 | -3.83 | 0.00 |
| RF-ARMA | ARIMA | -2.34 | 0.03 | 0.75 | 0.46 |
| RF-ARMA | SARIMA | -1.56 | 0.13 | 1.98 | 0.06 |
| RF-ARMA | SARIMAX | -2.73 | 0.01 | 0.10 | 0.92 |
| SVM-ARMA | RF | -4.54 | 0.00 | -4.28 | 0.00 |
| SVM-ARMA | XGB | -4.20 | 0.00 | -4.41 | 0.00 |
| SVM-ARMA | SVM | -4.61 | 0.00 | -4.88 | 0.00 |
| SVM-ARMA | ARIMA | -2.89 | 0.01 | 1.43 | 0.17 |
| SVM-ARMA | SARIMA | -2.74 | 0.01 | 0.17 | 0.87 |
| SVM-ARMA | SARIMAX | -3.03 | 0.01 | -2.74 | 0.01 |
| XGB-ARMA | RF | -4.46 | 0.00 | -4.23 | 0.00 |
| XGB-ARMA | XGB | -4.07 | 0.00 | -4.22 | 0.00 |
| XGB-ARMA | SVM | -4.47 | 0.00 | -4.75 | 0.00 |
| XGB-ARMA | ARIMA | -2.82 | 0.01 | -2.22 | 0.04 |
| XGB-ARMA | SARIMA | -2.52 | 0.02 | -0.50 | 0.04 |
| XGB-ARMA | SARIMAX | -2.99 | 0.01 | -3.03 | 0.01 |
